# Relationship Between Parapapillary Microvasculature Dropout and Visual Field Defect in Glaucoma: A Cross-Sectional OCTA Analysis

**DOI:** 10.3390/jcm14196936

**Published:** 2025-09-30

**Authors:** Fiorella Cuba-Sulluchuco, Carmen Mendez-Hernandez

**Affiliations:** 1 Ophthalmology Department, Hospital Clínico San Carlos, Institute of Health Research (IdISSC), 28040 Madrid, Spain; fiorella.cuba.4@gmail.com; 2 Department of Immunology, Ophthalmology and ORL, Ramon Castroviejo Institute for Ophthalmic Research, Complutense University of Madrid, 28040 Madrid, Spain

**Keywords:** OCTA, microvascular dropout, glaucoma, choroidal vascularization, peripapillary choroidal vascularity, peripapillary vessel density, visual field progression

## Abstract

**Background:** Glaucoma is a multifactorial optic neuropathy and the leading cause of irreversible blindness worldwide. Vascular mechanisms, including impaired perfusion of the optic nerve head, are increasingly recognized as contributors to disease progression. Optical coherence tomography angiography (OCTA) enables non-invasive assessment of retinal and choroidal microvasculature, including peripapillary microvasculature dropout (MvD), which may serve as a marker of glaucomatous damage. **Methods:** A cross-sectional case–control study was conducted, including patients with primary open-angle glaucoma (OAG) and healthy controls. All participants underwent a comprehensive ophthalmic evaluation and OCTA imaging using the PLEX Elite 9000 system. Peripapillary vessel density (pVD), flow index (pFI), peripapillary choroidal thickness (PCT), β-zone parapapillary atrophy (β-PPA), and choroidal vascular indices were measured. MvD was defined as the complete absence of microvasculature within the β-PPA boundary. Statistical analyses included univariate and multivariate regression models to examine variables associated with PCT and to assess the association between MvD and visual field mean defect (MD), as well as other glaucoma characteristics. ROC curve analysis was performed to evaluate the ability of MvD to discriminate between different levels of visual field defects. **Results:** A total of 87 eyes (41 glaucomatous, 46 controls) were analyzed. Glaucoma patients exhibited significantly lower pVD, pFI, PCT, and choroidal vascular indices compared to the controls. MvD was detected in 10 glaucomatous eyes and was associated with a larger β-PPA area, smaller choroidal luminal and stromal areas, and worse mean deviation (MD) values. Multivariate regression showed that the number of ocular hypotensive treatments and StructureIndex variables were significantly associated with PCT (adjusted R^2^ = 0.14). Logistic regression analysis identified MD, MD slope, and β-PPA area as variables significantly associated with the presence of MvD. ROC analysis showed that the presence of MvD had good discriminatory ability for visual field mean defects (MDs) (AUC = 0.77, 95% CI: 0.69–0.87; *p* = 0.005). **Conclusions:** Peripapillary MvD detected by OCTA is associated with reduced choroidal vascularity, increased β-PPA, and greater visual field deterioration in glaucoma patients. MvD may serve as a structural marker associated with functional deterioration in glaucoma patients.

## 1. Introduction

Glaucoma is the leading cause of irreversible blindness worldwide and is characterized as a progressive optic neuropathy with a multifactorial etiology, involving both mechanical and vascular mechanisms. According to the vascular theory, glaucomatous optic nerve damage results from insufficient blood perfusion, which may be influenced by elevated intraocular pressure (IOP) and other factors affecting ocular blood flow [1,2].

Optical coherence tomography angiography (OCTA) enables non-invasive imaging and structural assessment of both superficial and deep retinal vascular layers, making it a valuable tool for evaluating vascular alterations associated with glaucoma [3,4]. The demonstrated relationship between glaucoma progression and reduced blood flow from the short posterior ciliary arteries to the optic nerve head, along with decreased superficial peripapillary vessel density (pVD) in glaucoma patients, has led to growing interest in the evaluation of deeper peripapillary vascular layers [4,5,6].

Parapapillary microvasculature dropout (MvD), affecting both superficial and deep vascular layers, including the choroid, is increasingly recognized as a manifestation of glaucomatous damage in primary open-angle glaucoma (OAG) [7,8,9]. Moreover, reduced choroidal vascular density has been associated with glaucomatous progression [10]. The presence of MvD in the choroidal microvasculature has been linked not only to lower peripapillary vascular density [8,11] but also to decreased peripapillary choroidal thickness (PCT) [12]. Patients with lower pVD and increased MvD appear to be at greater risk of glaucomatous progression, independent of changes in the beta-zone parapapillary atrophy (β-PPA) [10,13].

The primary objective of this study is to identify the presence of parapapillary MvD and evaluate potential risk factors associated with its presence in patients with OAG.

The secondary objective is to assess choroidal thickness and vascularity in glaucoma patients versus healthy subjects and to analyze the relationship between MvD and β-PPA, pVD, and PCT.

## 2. Materials and Methods

### 2.1. Study Design and Participants

A cross-sectional case–control study was conducted, including glaucoma patients and healthy volunteers prospectively recruited and evaluated. Healthy subjects were selected from individuals attending the ophthalmology department for routine check-ups, with no family or personal history of glaucoma. All participants provided written informed consent prior to inclusion. The study adhered to Good Clinical Practice guidelines and was approved by the Ethics Committee of Hospital Clínico San Carlos (Protocol Nº 24/273-E).

The glaucoma group comprised patients diagnosed with OAG under regular follow-up at our institution. Diagnosis was confirmed through comprehensive ophthalmologic examination, including optic nerve head assessment, evaluation of the RNFL, and reproducible visual field defects.

Exclusion criteria for both groups included the following: ocular surgery within the previous six months, inflammatory eye disease, contact lens use, retinopathy or maculopathy, best corrected visual acuity (BCVA) < 20/32 in either eye, astigmatism > 3 diopters, or spherical refractive error > 5 diopters.

#### 2.1.1. Ophthalmic Examination

All participants underwent a standardized ophthalmic evaluation, including BCVA measurement; intraocular pressure (IOP) assessment using rebound tonometry (iCare, Tiolat Oy, Helsinki, Finland) and Goldmann applanation tonometry (Clement Clarke, Haag Streit, Harlow, UK); slit lamp biomicroscopy; non-dilated fundus examination; and visual field testing using the Octopus TOP G1 program (Octopus 600, Haag-Streit AG, Bern, Switzerland).

#### 2.1.2. OCTA Evaluation

Peripapillary vascular parameters, including choroidal analysis, were assessed using the OCTA PLEX Elite (Zeiss Meditec, Dublin, CA, USA) and the AngioPlex Elite 9000 algorithm (AngioPlex Elite 9000, Zeiss, Germany; version 0.9) via the Zeiss Advanced Imaging Nerwork Hub.

**Figure 1:**
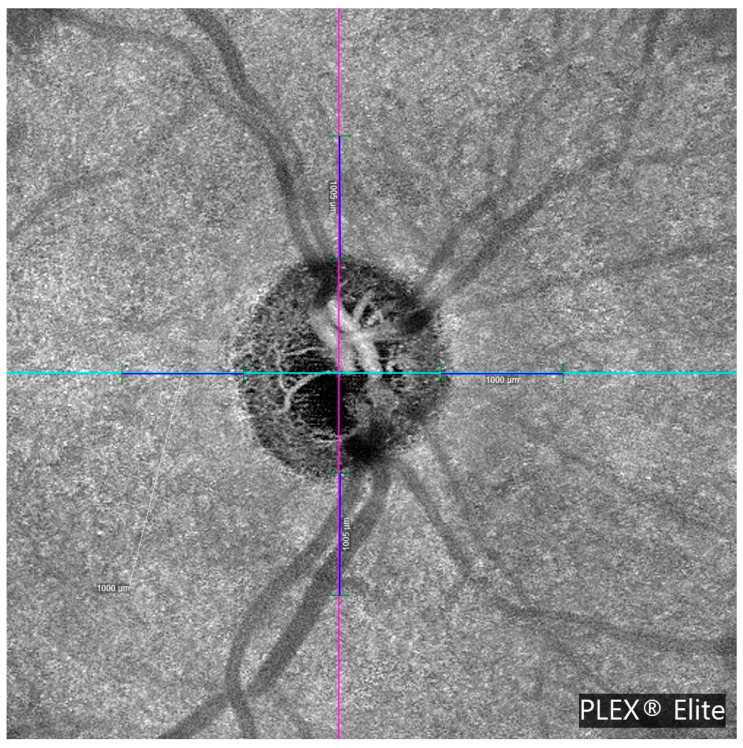
Optical coherence tomography angiography (OCTA) en face image of the optic nerve head (ONH) acquired using the PLEX^®^ Elite 9000 system. The image displays the central optic disc with visible neuroretinal rim and radiating peripapillary vasculature. Colored crosshairs indicate horizontal and vertical measurement axes, each labeled with a 1000 µm scale reference. This image was used to manually delineate the optic disc margin and β-zone parapapillary atrophy (β-PPA) boundaries for structural analysis and to select the measurement points for choroidal thickness.

**Figure 2:**
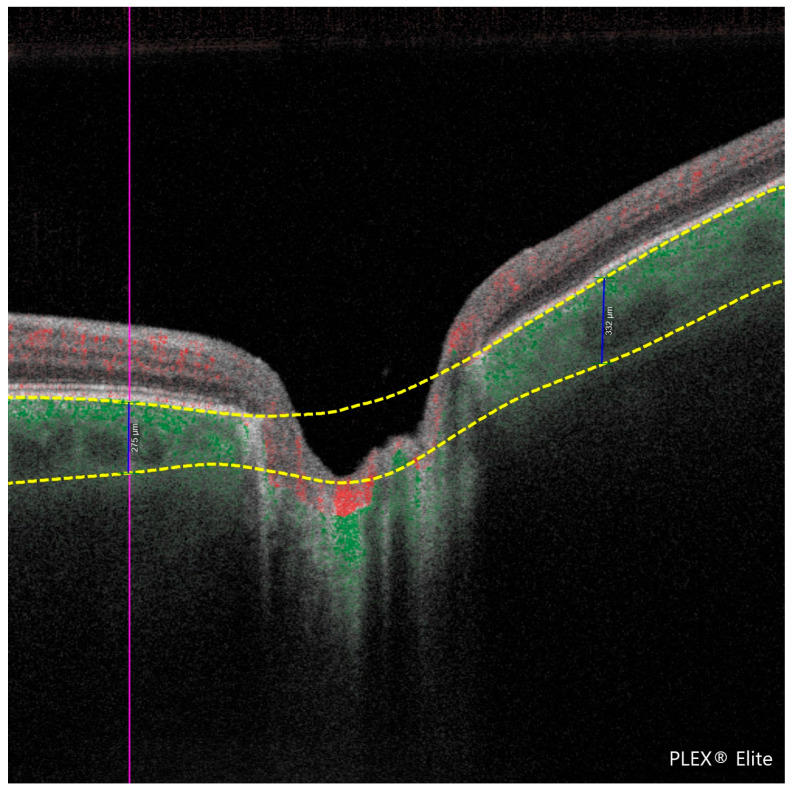
Cross-sectional optical coherence tomography (OCT) image of the retina acquired using the PLEX^®^ Elite 9000 system. The image illustrates the peripapillary choroidal thickness (PCT) measurements in two quadrants, with vertical blue lines indicating thickness values of 275 μm (**left**) and 332 μm (**right**). Red and yellow dashed lines delineate anatomical boundaries used for manual measurement, extending from the outer border of the retinal pigment epithelium to the inner scleral surface, at a fixed distance of 1000 μm from the optic disc margin.

PCT is the thickness of the choroid (the vascular layer beneath the retina) measured in the peripapillary region. In this study, PCT was manually measured at a fixed distance of 1000 μm from the optic disc margin in the four peripapillary quadrants (temporal, nasal, superior, and inferior) using high-resolution en face OCTA images. Measurement points were selected based on two anatomical landmarks, specifically from the outer border of the retinal pigment epithelium (RPE) to the inner scleral boundary. This standardized approach was applied consistently across all subjects to ensure reproducibility and minimize measurement bias. The methodology is illustrated in Figure 1 and Figure 2.

**Figure 3:**
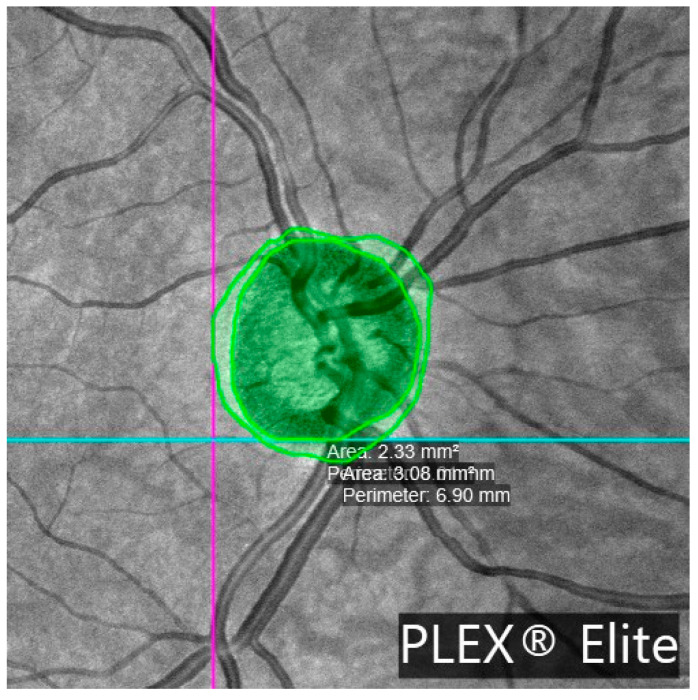
Optical coherence tomography angiography (OCTA) en face image of the optic nerve head (ONH) acquired using the PLEX^®^ Elite 9000 system. The image displays a manually delineated region of β-zone parapapillary atrophy (β-PPA), highlighted in green. The β-PPA area was calculated by subtracting the ONH area (2.33 mm^2^) from the total area (3.08 mm^2^). These parameters were used to assess structural alterations associated with microvascular dropout (MvD) in glaucomatous eyes.

β-zone parapapillary (β-PPA) was identified and quantified in the OCTA images. β-PPA was defined as the area between the optic disc margin (inner border of the peripapillary scleral ring) and the RPE atrophy line, characterized by visible sclera and prominent choroidal vessels. Manual delineation of the disc margin and β-PPA was performed on en face OCTA images using B-scan references. Figure 3 illustrates this process.

In all study participants, the presence of parapapillary microvascular loss (MvD) was assessed in the superficial and deep retinal vascular plexuses, choriocapillaris, and choroid. All vascular-layer images automatically provided by OCTA Angioplex were reviewed for this purpose.

Peripapillary vessel density (pVD), flow index (pFI), and RNFL thickness were automatically provided by the OCTA system. Manual measurements of PCT, β-PPA, and MvD detection were performed by the same examiner (FC), blinded to participant diagnosis. All choroidal images and the presence of MvD were also independently evaluated by a second examiner (CM).

Peripapillary vessel density is the proportion of the peripapillary area (the region surrounding the optic nerve head) occupied by blood vessels, as detected by OCTA. It is automatically calculated by the OCTA system as a percentage (from 0 to 1), reflecting the density of perfused microvasculature in the peripapillary retina. The peripapillary flow index quantifies the average flow signal intensity within the peripapillary region, as measured by OCTA. It is also automatically provided by the OCTA device, expressed as a proportion (0 to 1). Higher values indicate greater blood flow.

The choroidal vascularity index (CVI), luminal area (CLA), and stromal area (CSA) were measured from transverse OCTA en face choroidal scans in 6 × 6 mm images centered on the optic nerve head. Images were exported and analyzed using the ImageJ software (version 1.52q; NIH, Bethesda, Washington, DC, USA), applying pixel-to-micron conversion, automatic binarization, and skeletonization to quantify vascular flow signals. A rectangular region of interest (ROI) measuring 1.5 mm × 0.5 mm was defined in the temporal and nasal peripapillary regions, between the RPE-Bruch’s membrane complex and the sclera–choroidal junction. CVI was calculated as the percentage of the ROI area with a detectable flow signal. CLA was derived from the product of the ROI area and vascular flow index (VFI), and CSA was calculated by subtracting CLA from the ROI area [14,15].

All OCTA-derived measurements and clinical examination data were recorded in an anonymized Excel database.

#### 2.1.3. Statistical Analysis

Statistical analyses were performed using the IBM SPSS Statistics software (version 28 (28.0; IBM Corp., Somers, NY, USA).

Continuous variables were compared between groups using the independent samples Student’s *t*-test. Normality of the distributions was assessed via graphical inspection and Shapiro–Wilk tests. Homogeneity of variances was evaluated using Levene’s test, with Welch’s correction applied when necessary. Results were reported as *p*-values, mean differences, standard errors, and 95% confidence intervals.

Categorical variables were analyzed using Pearson’s chi-square test. For comparisons involving small sample sizes, non-normal distributions, or ordinal data, the nonparametric Mann–Whitney U test was used for continuous variables, and Fisher’s exact test was used for categorical variables. Results from nonparametric tests are presented as medians and interquartile ranges. Correlations between parameters were analyzed with Pearson’s correlation coefficient.

Interexaminer agreements regarding MvD measurements were assessed using the intraclass correlation coefficient (CCI).

Univariate linear regression was initially performed to explore the associations between global PCT (dependent variable) and the ophthalmic parameters that showed significant differences between the glaucoma and normal groups. To reduce redundancy and collinearity between two correlated structural parameters, RNFL thickness and pVD, we developed the combined variable StructureIndex. This index was calculated as the arithmetic mean of both measurements. The aim of combining these two variables into one was to increase the stability and interpretability of the model in multivariate and logistic regression analysis.

The following variables were included in the analysis: systemic arterial hypertension, β-PPA, number of ocular antihypertensive treatments, StructureIndex, MD of the visual field, and CLA. A multivariate linear regression model was subsequently constructed using the backward stepwise selection method, initially including all variables with significance (*p* < 0.05) or a trend toward significance (*p* < 0.10) in the univariate analysis. Assumptions of linearity, normality of residuals, and absence of multicollinearity were verified. Results are expressed as unstandardized β coefficients with 95% confidence intervals and *p*-values, with *p* < 0.05 considered statistically significant.

To identify the clinical and structural parameters associated with the presence of parapapillary MvD in glaucoma patients, binary logistic regression analysis was performed. The following variables were included in the model: age, number of ocular hypotensive treatments, IOP, pachymetry, MD, MD slope, RNFL progression slope, StructureIndex, β-PPA, PCT, and CVI.

To take into account the potential confounding effect of systemic arterial hypertension, it was included as a covariate in all regression models. Systemic arterial hypertension status was coded as a binary variable (yes/no) and included along with other clinical and structural parameters in both linear and logistic regression analysis. This approach allowed us to adjust for the potential impact of systemic arterial hypertension when assessing associations between glaucoma-related variables and vascular outcomes.

Statistically significant was defined as *p* < 0.05.

## 3. Results

A total of 60 subjects were prospectively enrolled, including 30 patients diagnosed with open-angle glaucoma and 30 healthy volunteers. Six participants, two glaucoma patients, and four healthy subjects were excluded due to the insufficient quality of OCTA imaging for evaluation. The final statistical analysis included 87 eyes: 41 eyes from 29 patients with established OAG and 46 eyes from 26 healthy controls.

No significant differences were found between groups regarding gender distribution. In the glaucoma group, 60.7% (17/28) were female, compared to 61.5% (16/26) in the control group (*p* = 0.951). The proportion of pseudophakic patients was similar between groups (61.5% in controls vs. 60.7% in glaucoma patients, *p* = 0.951). Hypercholesterolemia prevalence was comparable (19.2% in controls vs. 21.4% in glaucoma patients, *p* = 0.555), whereas hypertension was significantly more frequent in the glaucoma group (57.1% vs. 19.2%, *p* = 0.005). No participants with diabetes were included.

Table 1 summarizes demographic characteristics and the retinal nerve fiber layer (RNFL) thickness values obtained via OCTA PLEX Elite 9000. No significant differences were observed in age, refractive error, visual acuity, or central corneal thickness. Glaucoma patients had higher treated IOP (18.3 ± 4.7 vs. 16.2 ± 2.6 mmHg, *p* = 0.008), greater mean defects (MDs) (2.75 ± 4.0 vs. 1.14 ± 2.18 dB, *p* = 0.0013), and lower peripapillary RNFL thickness (74.7 vs. 81.5 μm, *p* < 0.001).

Among patients with glaucoma, 10.7% (3/28) had undergone filtering surgery, and 60.7% (17/28) were receiving topical antiglaucoma treatment. The mean number of medications per patient was 0.85 ± 0.16.

Table 2 presents peripapillary vascular density parameters, β-PPA measurements, PCT values across four peripapillary quadrants, and choroidal vascular indices (CVI, CLA, and CSA) in the temporal and nasal peripapillary sectors. Glaucoma patients exhibited significantly lower superficial vessel density (pVD 0.52 vs. 0.53, *p* = 0.006) and flow indices (pFI 0.34 vs. 0.37, *p* < 0.001).

β-PPA was present in 95.1% of glaucomatous eyes (39/41) and 78.3% of control eyes (36/46), *p* = 0.030. The β-PPA area was significantly larger in glaucoma patients (1.44 ± 1.36 vs. 0.88 ± 0.61 mm^2^, *p* = 0.039). Choroidal thickness was significantly reduced in glaucoma patients (162.99 ± 49.80 vs. 221.94 ± 76.79 μm, *p* = 0.007), along with smaller choroidal luminal areas (CLA: 0.26 ± 0.06 vs. 0.31 ± 0.07 mm^2^, *p* = 0.003) and stromal areas (CSA: 0.43 ± 0.12 vs. 0.50 ± 0.14 mm^2^, *p* = 0.014).

**Figure 4:**
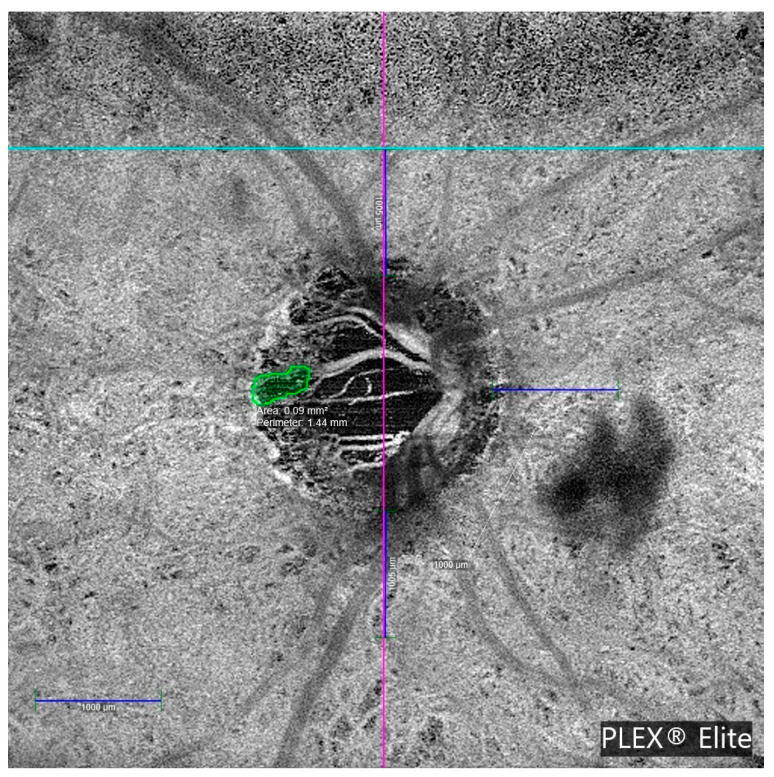
Optical coherence tomography angiography (OCTA) en face image acquired using the PLEX^®^ Elite 9000 system, showing a circular region of interest within the peripapillary area. Cyan and magenta crosshairs intersect at the center of the optic nerve head, providing spatial reference. Quantitative measurements include the total microvascular dropout (MvD) area (0.09 mm^2^) and perimeter (1.44 mm). Scale bars of 1000 µm are shown for calibration.

MvD was detected in 7 of the 29 OAG patients, with bilateral involvement in three cases, resulting in 10 affected eyes. No MvD was observed in healthy controls. Both examiners agreed on the determination of the presence of MvD in all cases. The interexaminer ICC for the MvD perimeter measurement was 0.979 (95% CI: 0.917–0.995; *p* < 0.001). Table 3 details the clinical characteristics of glaucoma patients with MvD. Most MvDs were located in deep vascular layers, specifically, the choriocapillaris and choroid. Only 2 of the 10 cases showed MvD in the superficial vascular plexus. All MvD-positive eyes exhibited β-PPA. Figure 4 illustrates a representative en face OCTA image showing MvD in a glaucomatous eye.

No significant differences in gender distribution were found between glaucoma patients with and without MvD (60% vs. 58%, *p* = 0.606). Similarly, pseudophakia rates were comparable (60% vs. 63%, *p* = 0.208. Two of the seven MvD-positive patients (28.6%) had undergone glaucoma surgery.

Table 4 summarizes demographic data for glaucoma patients with and without MvD, and Table 5 presents differences in the peripapillary vascular density parameters. MvD-positive eyes had significantly larger β-PPA areas (median [IQR]: 1.62 (1.17–1.91) vs. 0.87 (0.67–1.26) mm^2^, *p* = 0.011), smaller choroidal luminal areas (0.22 [0.17–0.26] vs. 0.27 [0.23–0.31] mm^2^, *p* = 0.015, and reduced choroidal stromal areas (0.30 [0.26–0.45] vs. 0.47 [0.35–0.55] mm^2^, *p* = 0.012.

Peripapillary vessel density showed a strong correlation with RNFL thickness (r = 0.715, *p* < 0.001). Sectorial analysis revealed the strongest correlation in the inferior peripapillary quadrant (r = 0.749, *p* < 0.001), followed by the superior (r = 0.700, *p* < 0.001), temporal (r = 0.654, *p* < 0.001), and nasal sectors (r = 0.419, *p* < 0.001). No correlation was found between pVD and choroidal thickness.

Univariate linear regression showed a significant negative association between the number of glaucoma medications and PCT (β = −24.94; 95% CI: −46.27 to −3.62; *p* = 0.022). StructureIndex showed a trend toward a positive association (*p* = 0.059). In the final multivariate model, both the number of medications (β = −23.37; 95% CI: −46.50 to −0.24; *p* = 0.048) and StructureIndex (β = 14.88; 95% CI: 0.32 to 29.45; *p* = 0.045) remained as independent variables significantly associated with PCT. The model explained 14% of the variability in global choroidal thickness (adjusted R^2^ = 0.14) (Table 6).

Binary logistic regression analysis revealed that the visual field MD index was significantly associated with the presence of MvD (OR = 1.283; 95% CI: 1.076–1.529; *p* = 0.005). The slope of MD progression was also significantly associated (OR = 3.198; 95% CI: 1.058–9.666; *p* = 0.039). β-PPA area showed a significant association (OR = 1.662; 95% CI: 1.012–2.729; *p* = 0.045). Other variables (systemic arterial hypertension, age, number of ocular hypotensive treatments, IOP, pachymetry, RNFL progression slope, StructureIndex, PCT, and CVI) did not show significant associations.

**Figure 5:**
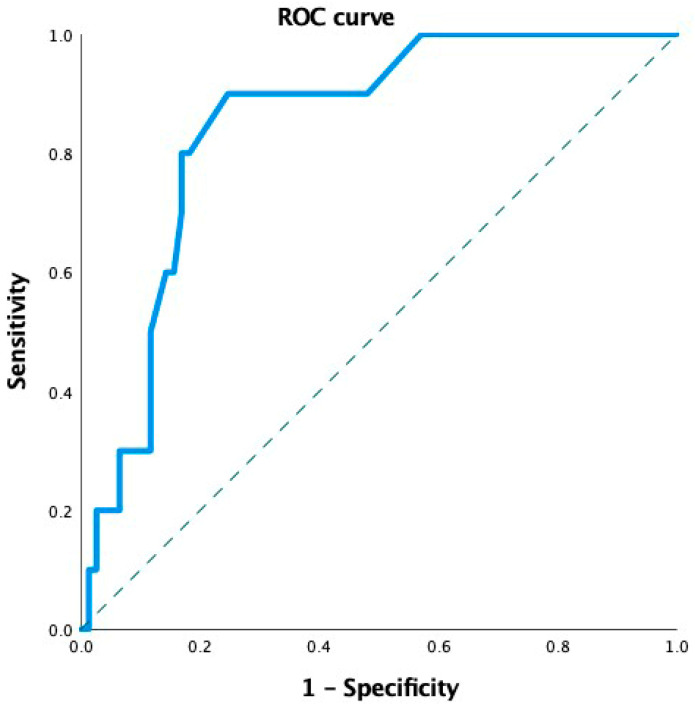
Receiver Operating Characteristic (ROC) curve showing the discriminative ability of parapapillary microvasculature dropout (MvD) presence to differentiate between different levels of visual field mean defects (MDs) in glaucoma patients. The area under the curve (AUC) was 0.77 (95% CI: 0.69–0.87; *p* = 0.005), indicating good discriminatory capacity.

Due to the limited number of MvD events (*n* = 10), a robust multivariate logistic regression model could not be constructed. Therefore, we assessed the ability of MvD to discriminate between different levels of MD impairment using Receiver Operating Characteristic (ROC) curve analysis. The area under the ROC curve (AUC) was 0.77 (95% CI: 0.69–0.87; *p* = 0.005), indicating good discriminatory capacity (Figure 5).

## 4. Discussion

This study identified parapapillary MvD in a subset of eyes with OAG, accounting for 24.4% of cases. The localization of MvD varied across retinal vascular layers, with a predominance in the choroid and choriocapillaris, suggesting deeper vascular compromise in glaucomatous pathology. These findings are consistent with previous reports indicating that MvD typically occurs within β-zone parapapillary atrophy and preferentially affects deeper microvascular layers [16].

Although dropout in the superficial and deep plexuses was less consistently observed, its absence does not exclude the presence of parapapillary MvD in other layers.

Secondary analysis revealed significant reductions in PCT, choroidal stromal and luminal areas, and pVD in glaucoma patients compared to healthy controls. These results support the hypothesis of vascular involvement in glaucomatous damage and align with prior studies emphasizing the diagnostic relevance of peripapillary vascular density [17,18,19].

The observed increase in β-PPA area and its association with parapapillary MvD further suggest a link between microvascular dropout and localized structural degeneration. Our results are consistent with previous studies that have reported a reduction in PCT in glaucomatous eyes, which may reflect vascular support dysfunction at the choroidal level, particularly in areas adjacent to the optic nerve head [20,21].

The inverse relationship between the number of ocular antiglaucomatous treatments and PCT, both in univariate and multivariate models, may reflect the impact of chronic intraocular pressure management on choroidal vascular dynamics. Additionally, the StructureIndex was positively associated with PCT in the multivariate model, indicating that structural integrity may contribute to maintaining choroidal architecture. Although variables such as β-PPA, choroidal luminal area, and visual field mean defect did not reach statistical significance, their inclusion highlights the multifactorial nature of choroidal changes in glaucoma. The adjusted R^2^ value (0.14) suggests that other unmeasured factors may contribute to the observed variability. Given that the sample size of the study was limited and that the multivariate linear regression models had insufficient statistical power to reliably detect real effects, it was decided to present the results individually using univariate linear regression analysis in addition to multivariate analysis.

A strong correlation between pVD and peripapillary RNFL thickness, particularly in the inferior and superior sectors, reinforces the interdependence between structural integrity and vascular perfusion in the optic nerve head [22,23,24].

The comparative analysis between glaucoma patients with and without parapapillary MvD revealed significant differences in specific clinical parameters. MvD-positive eyes exhibited more pathological MD values and greater visual field progression, suggesting a potential link between microvascular compromise and functional deterioration. Lower GAT IOP values in the parapapillary MvD group may reflect more advanced disease stages or treatment effects. No significant differences were found in RNFL thickness or its progressive thinning between groups, indicating that MvD is not independently associated with structural loss.

Further comparison of peripapillary superficial vessel and choroidal measurements revealed significantly lower pVD in the temporal and nasal sectors of MvD-positive eyes. These eyes also showed greater β-PPA, reduced choroidal luminal and stromal areas, and a trend toward thinner PCT. While not all differences reached statistical significance, the findings suggest localized microvascular compromise. Unlike previous studies that reported a direct relationship between MvD extent and reduced PCT or IOP [25,26,27,28], our study did not find significant differences in these variables, possibly due to the topographic focus of our analysis. On the other hand, we found that patients with MvD had significantly smaller choroidal luminal and stromal areas, suggesting that, although total choroidal thickness may not differ significantly, there are quantitative alterations in choroidal vascular perfusion in glaucoma eyes with MvD. Studies such as that by Lee et al. have already reported that MvD is associated with lower flow density and a pattern of localized choroidal thinning [27].

Importantly, all MvD-positive patients exhibited associated β-PPA, supporting the hypothesis that parapapillary atrophy represents a structurally vulnerable environment for vascular dropout. This observation is consistent with prior studies reporting an association between β-PPA extent and the presence of MvD [16,29]. Our results demonstrate that MvD is associated with worse MD values and greater MD progression, supporting its role as a structural marker of disease severity [13]. However, we did not observe a relationship between RNFL thickness changes and MvD, suggesting that choroidal MvD may not be independently associated with structural deterioration [30,31]. Our finding of no significant correlation between RNFL thinning and the presence of MvD is noteworthy, as it suggests that vascular dropout may represent a mechanism of glaucomatous functional progression that could be, at least in part, independent of structural damage. These findings support the hypothesis that vascular dysfunction, as reflected by MvD, may play a distinct and clinically relevant role in the progression of glaucomatous functional deterioration, independently of RNFL thinning.

Although superficial vascular density parameters (pVD and pFI) were lower in glaucoma patients, they did not differ significantly between those with and without parapapillary MvD. This indicates that OCTA-detected perfusion reduction may not reliably reflect deep microvascular compromise. The presence of parapapillary MvD was assessed in the superficial and deep vascular plexuses, choriocapillaris, and choroid. This strategy was chosen to provide a comprehensive assessment of microvascular compromise in glaucoma, as vascular dropout may occur at different depths and anatomical levels within the peripapillary region. Superficial and deep retinal vascular plexuses, choriocapillaris, and choroids are interconnected through a complex vascular network supplying the optic nerve head and peripapillary region mainly through the branches of the short posterior ciliary arteries. The pathological processes of glaucoma can affect these layers simultaneously or sequentially, as ischemic or degenerative processes affecting one layer may extend to or coexist in adjacent layers due to a shared or closely related vascular supply, as well as anatomical and functional interconnections. Therefore, simultaneous assessment of all these layers allows for a more accurate characterization of the extent and pattern of microvascular dropout and may help to elucidate whether vascular compromise is focal or diffuse.

Our findings support the theory that the combined involvement of the retinal and choroidal microvasculature is associated with more severe functional impairment in glaucoma. Nevertheless, the association between MvD and worse MD values and greater visual field progression suggests that deeper vascular involvement may be more pronounced in advanced disease stages, as previously described [32].

To explore the possible influence of systemic arterial hypertension on peripapillary choroidal thickness (PCT) and the presence of MvD in glaucoma patients, we performed a stratified analysis according to systemic arterial hypertension status, as presented in Supplementary Table S1. This table shows the PCT measurement and the presence or absence of MvD in both the control and glaucoma groups, separated by the presence or absence of systemic arterial hypertension. No statistically significant differences in PCT values were observed between hypertensive and non-hypertensive subjects, either in the control group (*p* = 0.285) or in glaucoma patients (*p* = 0.202). Similarly, the prevalence of MvD did not differ significantly between hypertensive and non-hypertensive patients with glaucoma (22% vs. 28%, *p* = 0.936).

Furthermore, univariate linear regression analysis revealed that systemic arterial hypertension was not significantly associated with PCT (B = −4.24, 95% CI: −37.15 to 28.67, *p* = 0.798). Similarly, binary logistic regression analysis showed no significant association between systemic arterial hypertension and the presence of MvD (B = −0.325, 95% CI: 0.173–3.019, *p* = 0.722). These findings suggest that, within the limitations of our sample size, systemic arterial hypertension does not have a relevant impact on PCT or the presence of MvD in our study group.

Limitations of this study include the relatively small sample size, particularly in the MvD subgroup, which may have limited the statistical power to detect significant differences in some comparisons.

Regarding statistical power and sample size limitations, although a significant association between the MD index and the presence of microvasculature dropout (MvD) was found, we acknowledge that the number of glaucomatous eyes with MvD included in the analysis (*n* = 10) is limited. A post hoc power analysis was conducted to address the possibility of overfitting and false-positive results in both the regression models and the ROC curve analysis. Using the observed odds ratio (OR = 1.283), a significance level of α = 0.05, and the proportion of MvD-positive cases (11.5%), the estimated statistical power was about 71%, indicating a moderate ability to detect true effects. Ideally, an a priori power analysis would have required a sample size of at least 150–180 eyes to achieve 80% power under similar assumptions. This could be considered a methodological limitation and underscores the need for future studies with larger samples to validate our findings.

The cross-sectional design precludes causal inference. Manual quantification of PCT and β-PPA may be subject to interobserver variability, although all measurements were performed by a single examiner using high-resolution en face OCTA images.

Strengths include the simultaneous sectoral evaluation of multiple vascular and structural parameters using a single high-resolution OCTA platform. The use of a composite index (StructureIndex) improved model robustness and reduced collinearity.

The StructureIndex is a new combined variable that integrates structural and vascular information from RNFL thickness and pVD. Both measures are correlated and show the complementary characteristics of optic nerve head morphology and vascularization assessed by SD-OCT and OCTA imaging. Its inclusion in this study was mainly methodological, addressing collinearity and sample size limitations.

This index did not demonstrate a statistically significant advantage over pVD or RNFL thickness in discriminating between glaucoma patients with or without MvD.

The StructureIndex had an AUROC value of 0.681 (SE 0.080; 95% CI: 0.524–0.838), compared with 0.519 (SE 0.101; 95% CI: 0.321–0.717) for pVD and 0.560 (SE 0.109; 95% CI: 0.347–0.773) for peripapillary RNFL thickness. A statistical comparison of the ROC curves using a z-test showed no significant differences between the StructureIndex and pVD (z = 1.257, *p* = 0.21) or between the StructureIndex and RNFL thickness (z = 0.895, *p* = 0.37).

For this reason, although this combined parameter showed a statistically significant association with PCT in our multivariate model, we acknowledge that this index has not been validated in previous studies, so these results should be interpreted with caution.

Its validation in future studies and longitudinal designs would be necessary to verify its usefulness.

This study was conducted at a single center with a relatively homogeneous population, which may limit the generalizability of our findings. It is possible that the association observed between MvD and glaucomatous progression may differ in populations with different ethnic, genetic, or systemic risk profiles. For example, vascular autoregulation, susceptibility to microvascular compromise, and optic nerve head structural characteristics may vary among different demographic groups. Multicenter studies involving more diverse populations are needed to validate our results and to determine whether MvD performs similarly as a marker of functional progression in different clinical contexts.

Prospective recruitment under uniform imaging and clinical protocols minimized bias, and manual validation of OCTA-derived parameters by a blinded examiner enhanced internal consistency. These methodological aspects strengthen the reliability of our findings and provide a basis for future longitudinal studies aimed at confirming the prognostic value of choroidal MvD in glaucoma progression.

In conclusion, parapapillary MvD in deep vascular layers is associated with reduced choroidal vascular indices, greater β-parapapillary atrophy, and visual field deterioration.

Peripapillary choroidal thickness and vascularization are lower in patients with glaucoma. Glaucomatous eyes with OCTA-conformed parapapillary choroidal dropout show lower choroidal vascular indices than those without dropout. MvD is associated with the worsening of mean defects, suggesting its possible role as a marker of increased risk of functional impairment.

## Figures and Tables

**Table 1:** Demographic and clinical data of study participants.

		Mean	Standard Deviation	* p-Value
Age (yr)	Control	70.27	6.98	0.093
	Glaucoma	70.18	9.10	
Sphere	Control	0.18	1.51	0.486
	Glaucoma	0.24	1.48	
Astigmatism	Control	−0.86	0.68	0.216
	Glaucoma	−1.00	0.92	
BCVA	Control	0.84	0.22	0.158
	Glaucoma	0.79	0.21	
iCare IOP (mmHg)	Control	16.54	2.77	0.161
	Glaucoma	17.29	4.05	
GAT IOP (mmHg)	Control	16.20	2.63	0.008
	Glaucoma	18.27	4.70	
Antiglaucoma medication (n)	Control	N/A	N/A	N/A
	Glaucoma	0.85	0.16	N/A
Pachymetry (μm)	Control	541.8	34.61	0.333
	Glaucoma	537.98	45.81	
Mean Defect (dB)	Control	1.14	2.18	0.013
	Glaucoma	2.75	4.00	
Average RNFL thickness (μm)	Control	81.50	6.84	<0.001
	Glaucoma	74.69	11.32	
RNFL temporal sector (μm)	Control	62.94	8.36	0.067
	Glaucoma	59.57	11.84	
RNFL superior sector (μm)	Control	97.02	14.10	0.032
	Glaucoma	90.36	18.24	
RNFL nasal sector (μm)	Control	64.29	7.55	0.114
	Glaucoma	62.26	8.05	
RNFL inferior sector (μm)	Control	104.50	11.27	<0.001
	Glaucoma	89.14	18.22	
GH Average RNFL thickness (μm)	Control	94.30	8.84	<0.001
	Glaucoma	85.69	14.90	
GH temporal RNFL sector (μm)	Control	70.62	10.31	0.122
	Glaucoma	67.38	14.75	
GH superotemporal RNFL sector (μm)	Control	125.45	21.38	0.007
	Glaucoma	109.99	33.59	
GH superonasal RNFL sector (μm)	Control	104.07	29.12	0.210
	Glaucoma	99.18	26.94	
GH nasal RNFL sector (μm)	Control	79.29	11.82	0.045
	Glaucoma	74.99	11.57	
GH inferotemporal RNFL sector (μm)	Control	144.42	20.47	<0.001
	Glaucoma	118.33	34.60	
GH inferonasal RNFL sector (μm)	Control	111.79	23.27	0.003
	Glaucoma	96.82	22.26	

GH: Garway–Heath map sectors; GAT: Goldmann applanation tonometry; BCVA: best corrected visual acuity; RNFL: retinal nerve fiber layer. * Statistical comparisons were performed using Student’s *t*-test. *p*-Values are reported for comparisons between control and glaucoma groups.

**Table 2:** Peripapillary vascular indices of all study participants.

		Mean	Standard Deviation	* *p*-Value
Average flow index (%)	Control	0.37	0.03	<0.001
	Glaucoma	0.34	0.04	
Flow index in temporal sector	Control	0.38	0.03	<0.001
	Glaucoma	0.34	0.04	
Flow index in superior sector	Control	0.37	0.03	<0.001
	Glaucoma	0.33	0.04	
Flow index in nasal sector	Control	0.37	0.03	<0.001
	Glaucoma	0.33	0.04	
Flow index in inferior sector	Control	0.37	0.03	<0.001
	Glaucoma	0.33	0.04	
Average vessel density (%)	Control	0.53	0.02	0.006
	Glaucoma	0.52	0.03	
Vessel density in temporal sector (%)	Control	0.55	0.02	0.082
	Glaucoma	0.54	0.04	
Vessel density in superior sector (%)	Control	0.53	0.03	0.021
	Glaucoma	0.51	0.04	
Vessel density in nasal sector (%)	Control	0.51	0.02	0.239
	Glaucoma	0.52	0.03	
Vessel density in inferior sector (%)	Control	0.55	0.02	<0.001
	Glaucoma	0.52	0.04	
Beta-parapapillary atrophy (PPA) measurement (mm^2^)	Control	0.88	0.61	0.039
	Glaucoma	1.40	1.36	
Peripapillary choroidal thickness (μm)	Control	221.94	76.79	<0.001
	Glaucoma	162.99	49.80	
Temporal choroidal thickness (μm)	Control	246.35	96.59	<0.001
	Glaucoma	166.39	60.55	
Nasal choroidal thickness (μm)	Control	214.33	75.05	0.002
	Glaucoma	170.88	57.67	
Superior choroidal thickness (μm)	Control	237.8	90.76	<0.001
	Glaucoma	177.05	64.32	
Inferior choroidal thickness (μm)	Control	189.28	65.23	<0.001
	Glaucoma	137.66	42.24	
Choroidal vascularity index	Control	0.80	0.08	0.065
	Glaucoma	0.77	0.08	
Choroidal luminal area (mm^2^)	Control	0.31	0.07	0.003
	Glaucoma	0.26	0.07	
Choroidal stromal area (mm^2^)	Control	0.50	0.14	0.014
	Glaucoma	0.43	0.12	

* Statistical comparisons were performed using Student’s *t*-test. *p*-Values are reported for comparisons between control and glaucoma groups and are shown in the Control row. Flow index and vessel density values are expressed as proportions ranging from 0 to 1.

**Table 3:** Patients with peripapillary dropout (pMvD). Localization of dropout and demographic characteristics of glaucoma patients with peripapillary MvD.

		Localization of Dropout												
	Diagnostic	Eye with MvD Detected	Superficial Vascular Plexus	Deep Vascular Plexus	Choriocapillaris	Choroid	Parapapillary Atrophy (mm^2^)	Age (yr)	Gender	Glaucoma Medications (*n*)	Glaucoma Surgery (*n*)	Hypercholesterolemia	Diabetes Mellitus	Systemic Arterial Hypertension
Patient 1	Glaucoma	Right	-	-	-	Yes	2.49	73	Male	0	0	No	No	Yes
Patient 1	Glaucoma	Left	-	-	Yes	Yes	6.27	73	Male	0	0	No	No	Yes
Patient 2	Glaucoma	Right	Yes	-	Yes	Yes	1.56	71	Male	1	0	No	No	No
Patient 2	Glaucoma	Left	-	-	-	Yes	1.27	71	Male	1	0	No	No	No
Patient 3	Glaucoma	Right	Yes	-	-	Yes	1.33	78	Female	1	0	No	No	No
Patient 3	Glaucoma	Left	-	-	-	Yes	0.83	78	Female	1	0	No	No	No
Patient 4	Glaucoma	Right	-	-	-	Yes	1.68	81	Female	0	0	No	No	Yes
Patient 5	Glaucoma	Left	-	-	Yes	Yes	1.72	77	Female	1	1	No	No	No
Patient 6	Glaucoma	Right	-	-	-	Yes	0.86	60	Female	1	0	Yes	No	Yes
Patient 7	Glaucoma	Right	-	-	-	Yes	1.68	74	Female	0	1	No	No	Yes

Localization of peripapillary microvascular dropout (pMvD) was assessed across vascular layers. Systemic conditions and ocular history are reported per eye. “Yes/No” indicates presence or absence of condition. Empty cells indicate no dropout detected in the corresponding layer.

**Table 4:** Demographic characteristics of glaucoma patients with and without peripapillary dropout (MvD).

	MvD	Median	IQR	* *p*-Value
Age (yr)	Yes	73.50	69.50; 78.00	0.604
	No	71.00	63.00; 77.00	
Sphere	Yes	0.13	−2.81; 1.75	0.482
	No	0.50	−0.25; 1.00	
Astigmatism	Yes	−1.25	−2.31; −0.69	0.017
	No	−0.75	−1.00; −0.75	
BCVA	Yes	0.85	0.78; 0.90	0.893
	No	0.80	0.70; 1.00	
iCare IOP (mmHg)	Yes	14.50	13.75; 19.00	0.075
	No	16.00	15.0; 22.00	
GAT IOP (mmHg)	Yes	14.50	12.75; 19.25	0.036
	No	18.0	15.00; 23.00	
Antiglaucoma medications (n)	Yes	1	0; 1	0.314
	No	1	0; 2	
Pachymetry (μm)	Yes	523.50	510.25–601.50	0.569
	No	526.00	510.25; 558.50	
Mean defect (dB)	Yes	3.95	2.95; 7.68	0.004
	No	0.50	−0.30; 3.30	
Average RNFL thickness (μm)	Yes	73.80	66.48; 86.49	0.560
	No	69.42	63.43; 83.47	
RNFL temporal sector (μm)	Yes	65.74	55.35; 72.13	0.081
	No	53.69	49.14; 67.65	
RNFL superior sector (μm)	Yes	101.11	77.70; 109.44	0.314
	No	84.56	74.27; 106.82	
RNFL nasal sector (μm)	Yes	61.54	60.02; 66.46	0.687
	No	62.13	57.19; 66.23	
RNFL inferior sector (μm)	Yes	79.85	58.25; 112.41	0.622
	No	89.56	79.78; 105.83	
GH Average RNFL thickness (μm)	Yes	86.31	74.82; 99.52	0.754
	No	79.54	72.81; 98.05	
GH RNFL temporal sector (μm)	Yes	72.36	63.72; 81.55	0.127
	No	60.34	53.29; 77.38	
GH superotemporal RNFL sector (μm)	Yes	112.50	84.73; 151.51	0.482
	No	102.67	84.19; 123.15	
GH superonasal RNFL sector (μm)	Yes	106.83	75.56; 140.83	0.520
	No	90.48	76.26; 115.90	
GH nasal RNFL sector (μm)	Yes	74.06	67.98; 80.86	0.731
	No	74.76	69.43; 81.82	
GH inferotemporal RNFL sector (μm)	Yes	103.28	59.00; 157.65	0.580
	No	121.83	97.99; 149.39	
GH inferonasal RNFL sector (μm)	Yes	101.43	65.02; 126.69	0.777
	No	99.09	82.26; 112.05	
Mean Defect change (dB)	Yes	0.20	0; 1.40	0.025
	No	−0.10	−0.60; 0.18	
RNFL change (μm)	Yes	−0.67	−2.10; −0.20	0.262
	No	−0.20	−1.20; 0	
Mean follow-up (years)	Yes	13.00	7.50; 17.00	0.177
	No	7	5; 13	

MvD: peripapillary microvascular dropout; GH: Garway–Heath map sectors; GAT: Goldmann applanation tonometry; BCVA: best corrected visual acuity; RNFL: retinal nerve fiber layer. Values are expressed in median and interquartile range (IQR). * The *p*-value corresponds to the exact two-tailed significance calculated using the Mann–Whitney U test. *p*-Values are shown in the “Yes” row only.

**Table 5:** Differences in choroidal measurements between glaucoma patients with and without peripapillary MvD.

	Dropout (MvD)	Median	IQR	* *p*-Value
**Average flow index (%)**	Yes	0.32	0.31; 0.36	0.345
	No	0.34	0.32; 0.37	
**Flow index in temporal sector**	Yes	0.33	0.31; 0.37	0.731
	No	0.35	0.32; 0.38	
**Flow index in superior sector**	Yes	0.32	0.31; 0.36	0.393
	No	0.34	0.31; 0.35	
**Flow index in nasal sector**	Yes	0.31	0.30; 0.35	0.235
	No	0.33	0.31; 0.37	
**Flow index in inferior sector**	Yes	0.31	0.30; 0.35	0.376
	No	0.34	0.30; 0.36	
**Average vessel density (%)**	Yes	0.53	0.51; 0.54	0.360
	No	0.52	0.51; 0.53	
**Vessel density in temporal sector (%)**	Yes	0.55	0.54; 0.58	0.042
	No	0.53	0.50; 0.56	
**Vessel density in superior sector (%)**	Yes	0.52	0.48; 0.55	0.709
	No	0.52	0.49; 0.54	
**Vessel density in nasal sector (%)**	Yes	0.53	0.51; 0.54	0.049
	No	0.51	0.50; 0.52	
**Vessel density in inferior sector (%)**	Yes	0.51	0.47; 0.53	0.190
	No	0.53	0.51; 0.54	
**Beta-zone parapapillary atrophy (BPPA) (mm^2^)**	Yes	1.62	1.17; 1.91	0.011
	No	0.87	0.67; 1.26	
**Peripapillary choroidal thickness (μm)**	Yes	137.62	116.44; 189.44	0.329
	No	161.50	129.25; 201.25	
**Temporal choroidal thickness (μm)**	Yes	150.00	114.75; 261.0	0.917
	No	158.00	115.0; 201.0	
**Nasal choroidal thickness (μm)**	Yes	124.50	113.25; 193.50	0.180
	No	167.00	141.0; 215.0	
**Superior choroidal thickness (μm)**	Yes	142.50	126.75; 230.0	0.560
	No	173.00	124.0; 207.0	
**Inferior choroidal thickness (μm)**	Yes	102.00	95.25; 150.50	0.119
	No	136.00	115.0; 167.0	
**Choroidal vascular index (%)**	Yes	0.80	0.73; 0.83	0.345
	No	0.75	0.72; 0.80	
**Choroidal luminal area (mm^2^)**	Yes	0.22	0.17; 0.26	0.015
	No	0.27	0.23; 0.31	
**Choroidal stromal area (mm^2^)**	Yes	0.30	0.26; 0.45	0.012
	No	0.47	0.35; 0.55	

BPPA: beta-zone parapapillary atrophy; MvD: peripapillary dropout; IQR: interquartile range. Values are expressed in median and interquartile range (IQR). * The *p*-value corresponds to the exact two-tailed significance calculated using the Mann–Whitney U test. *p*-Values are shown in the “Yes” row only. Flow index and vessel density are expressed as proportions ranging from 0 to 1.

**Table 6:** Results of the univariate and multivariate regression models in evaluating factors that may influence peripapillary choroidal thickness.

Variable	Model	B	95% CI (Min, Max)	*p*	Adjusted R^2^
Systemic arterial hypertension	Univariate	−4.24	(−37.15, 28.67)	0.798	-
Number of ocular antihypertensive treatments	Univariate	−24.90	(−46.27, −3.62)	0.022	-
StructureIndex	Univariate	10.64	(−0.44, 21.72)	0.059	-
Beta peripapillary atrophy (mm^2^)	Univariate	−8.09	(−23.27, 7.1)	0.292	-
Choroidal luminal area (mm^2^)	Univariate	48.10	(−174.29, 270.56)	0.668	-
Mean defect (dB)	Univariate	−2.45	(−7.21, 2.32)	0.310	-
Number of ocular antihypertensive treatments	Multivariate	−23.37	(−46.5, −0.24)	0.048	0.14
StructureIndex	Multivariate	14.88	(0.32, 29.45)	0.045	0.14

Note: B = regression coefficient; CI = confidence interval; *p* = *p*-value. Adjusted R^2^ is only reported for multivariate models. Negative B values indicate an inverse association with peripapillary choroidal thickness.

## Data Availability

The data presented in this study are available upon request from the corresponding author due to the privacy of the research.

## Supplementary Materials

The following supporting information can be downloaded at https://www.mdpi.com/article/10.3390/jcm14196936/s1. Supplementary Table S1. Peripapillary choroidal thickness (PCT) and prevalence of parapapillary microvasculature dropout (MvD) in glaucoma and control groups, stratified by the presence of systemic arterial hypertension. Data are presented as mean ± standard deviation for PCT and as percentage (n/N) for MvD prevalence. Statistical com-parisons were performed using Student’s *t*-test for continuous variables and Fisher’s exact test for categorical vari-ables. No statistically significant differences were observed in PCT or MvD prevalence between hypertensive and non-hypertensive subjects in either group (*p* > 0.05).

## Abbreviations

OCTA	Optical Coherence Tomography Angiography
MvD	Microvasculature Dropout
OAG	Open-Angle Glaucoma
pVD	Peripapillary Vessel Density
pFI	Peripapillary Flow Index
PCT	Peripapillary Choroidal Thickness
β-PPA	β-zone Parapapillary Atrophy
RNFL	Retinal Nerve Fiber Layer
BCVA	Best Corrected Visual Acuity
IOP	Intraocular Pressure
CVI	Choroidal Vascularity Index
CLA	Choroidal Luminal Area
CSA	Choroidal Stromal Area
MD	Mean Defect
ROC	Receiver Operating Characteristic
AUC	Area Under the Curve
SD-OCT	Spectral-Domain Optical Coherence Tomography
ROI	Region of Interest
VFI	Vascular Flow Index
StructureIndex	Composite Index Combining pVD and RNFL Thickness
