# Supplementary material for: Relationship Between Parapapillary Microvasculature Dropout and Visual Field Defect in Glaucoma: A Cross-Sectional OCTA Analysis
Source: J Clin Med. 2025 Sep 30;14(19):6936. doi: 10.3390/jcm14196936 (PMC12524885; doi:10.3390/jcm14196936)
Supplement: Supplementary file 1 [file jcm-14-06936-s001.zip › jcm-3861994-supplementary.pdf]

Supplementary Table S1

Supplementary Table S1. Peripapillary choroidal thickness (PCT) and prevalence of parapapillary microvasculature dropout (MvD) in glaucoma and control groups, stratified by the presence of systemic arterial hypertension. Data are presented as mean ± standard deviation for PCT and as percentage (n/N) for MvD prevalence. Statistical comparisons were performed using Student's t-test for continuous variables and Fisher's exact test for categorical variables. No statistically significant differences were observed in PCT or MvD prevalence between hypertensive and non-hypertensive subjects in either group ($p > 0.05$).

Systemic Hypertension	Group	PCT Mean ± SD (µm)	*p*-value (glaucoma vs. control)*	*p*-value (vs. other hypertension status)#	MvD Prevalence
Yes	Control	155.08 ± 31.81	—	0.180	—
Yes	Glaucoma	209.36 ± 89.00	0.079	_	22% (5/23)
No	Control	197.28 ± 69.44	—	0.077	—
No	Glaucoma	186.74 ± 59.88	0.291	_	28% (5/18)

Abbreviations:

PCT: Peripapillary Choroidal Thickness

MvD: Microvasculature Dropout

SD: Standard Deviation

n/N: Number of cases with MvD / Total number of eyes

µm: Micrometers
